# Supplementary figures and images for: Evolutionary Events Promoted Polymerase Activity of H13N8 Avian Influenza Virus
Source: Viruses. 2024 Feb 21;16(3):329. doi: 10.3390/v16030329 (PMC10975323; doi:10.3390/v16030329)

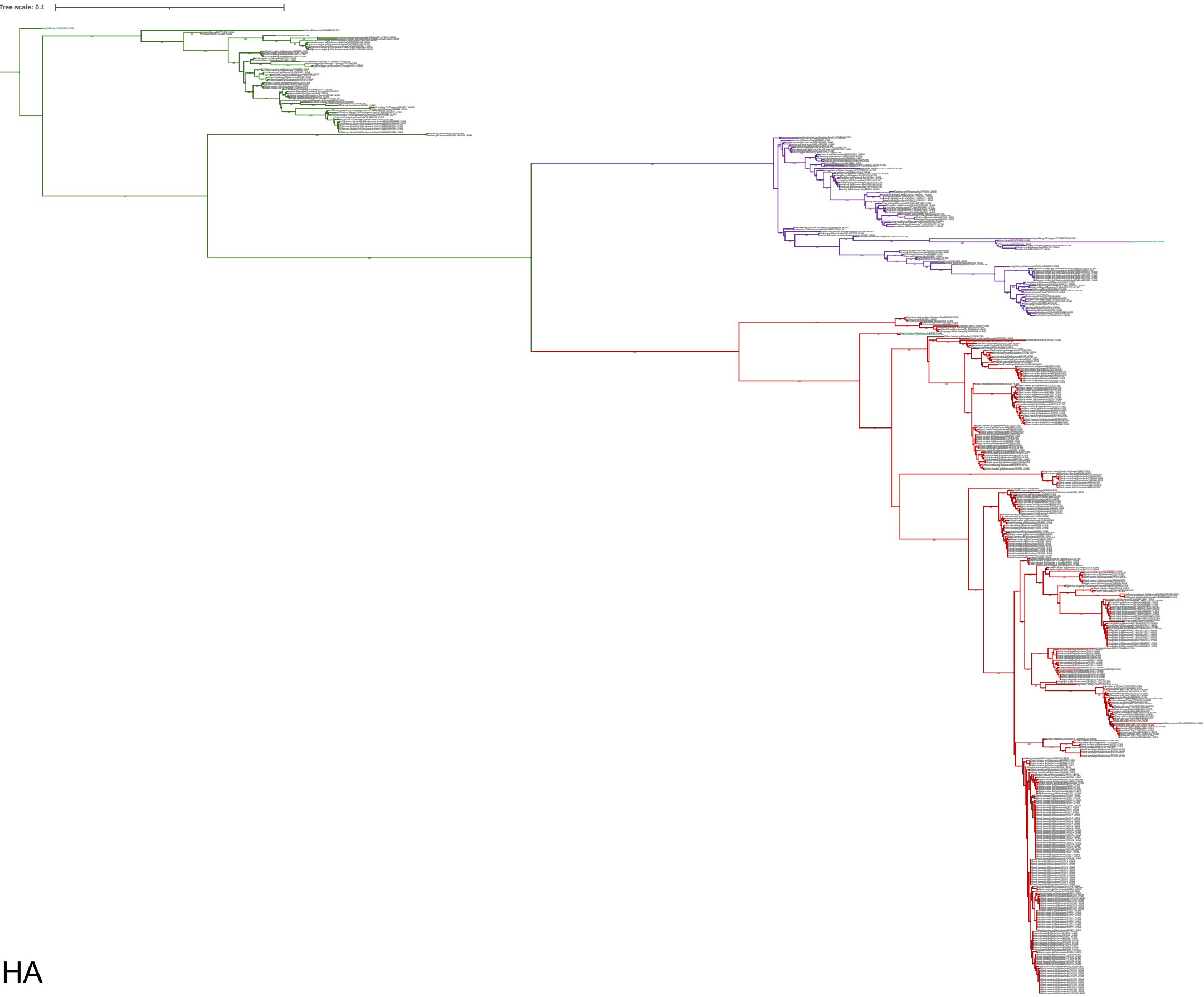

HA

Supplement: Supplementary file 1 [file viruses-16-00329-s001.zip › Supplementary Figure S2.pdf]

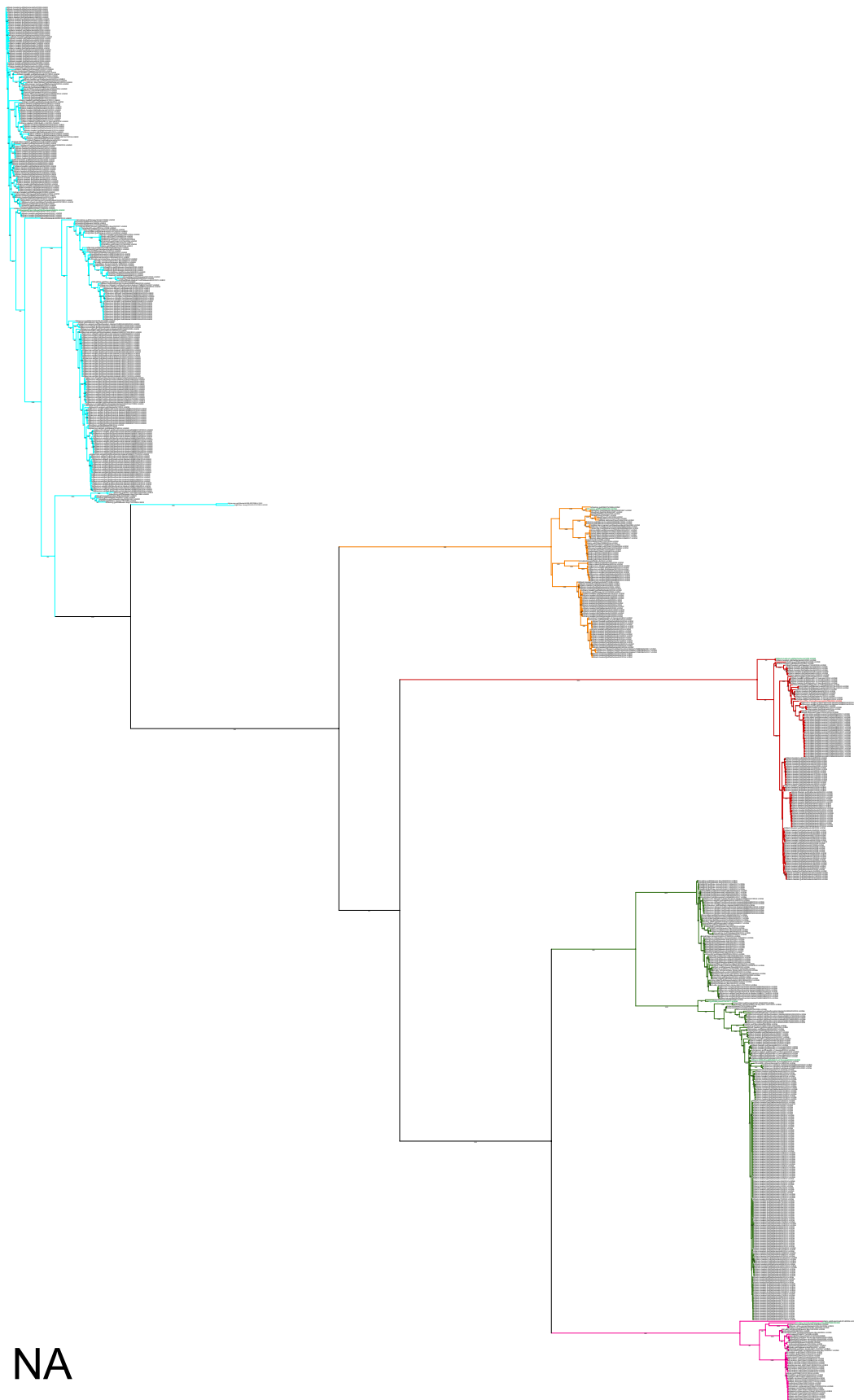

NA

Supplement: Supplementary file 1 [file viruses-16-00329-s001.zip › Supplementary Figure S3.pdf]

Tree scale: 0.1

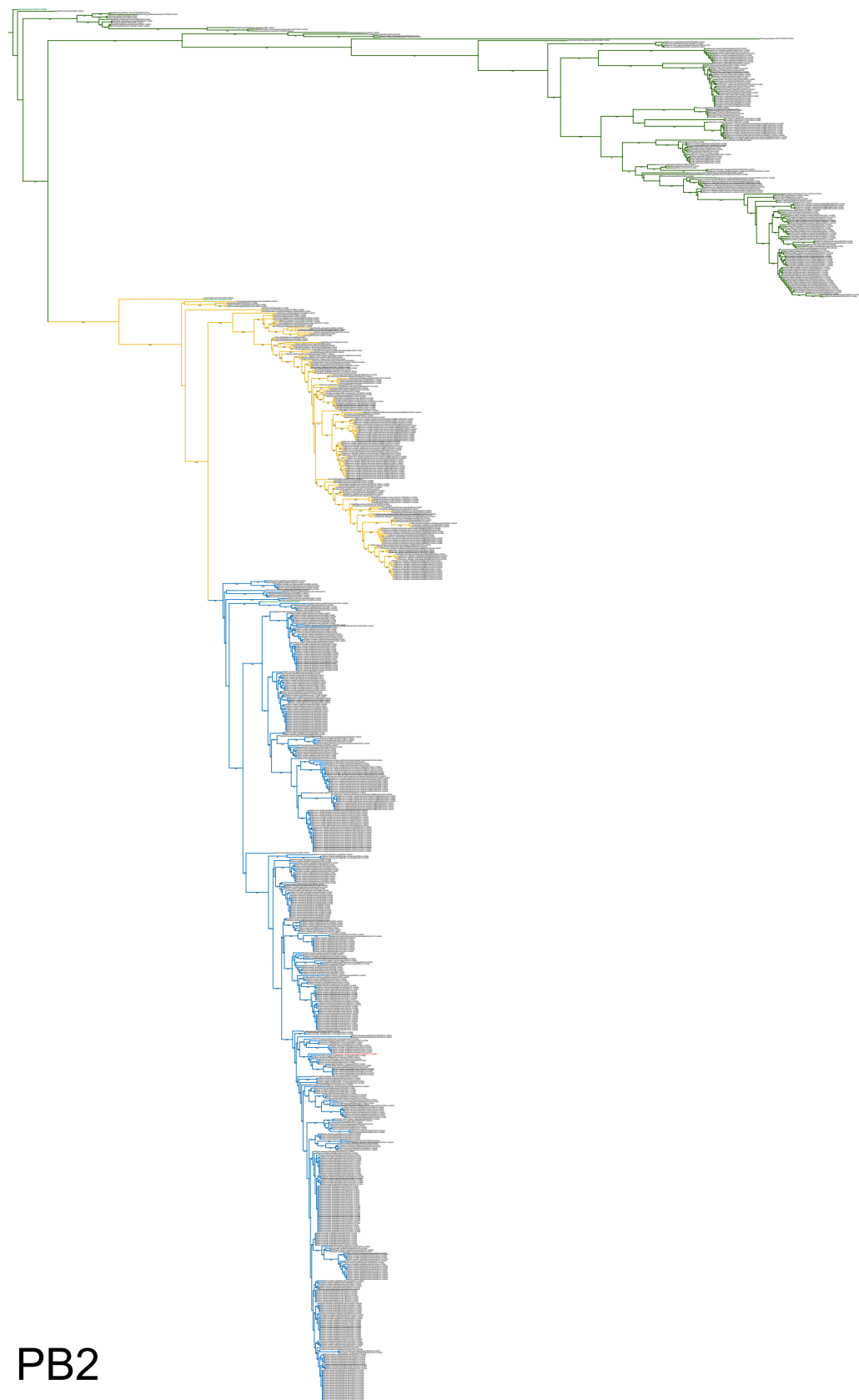

PB2

Supplement: Supplementary file 1 [file viruses-16-00329-s001.zip › Supplementary Figure S4.pdf]

Tree scale: 0.1

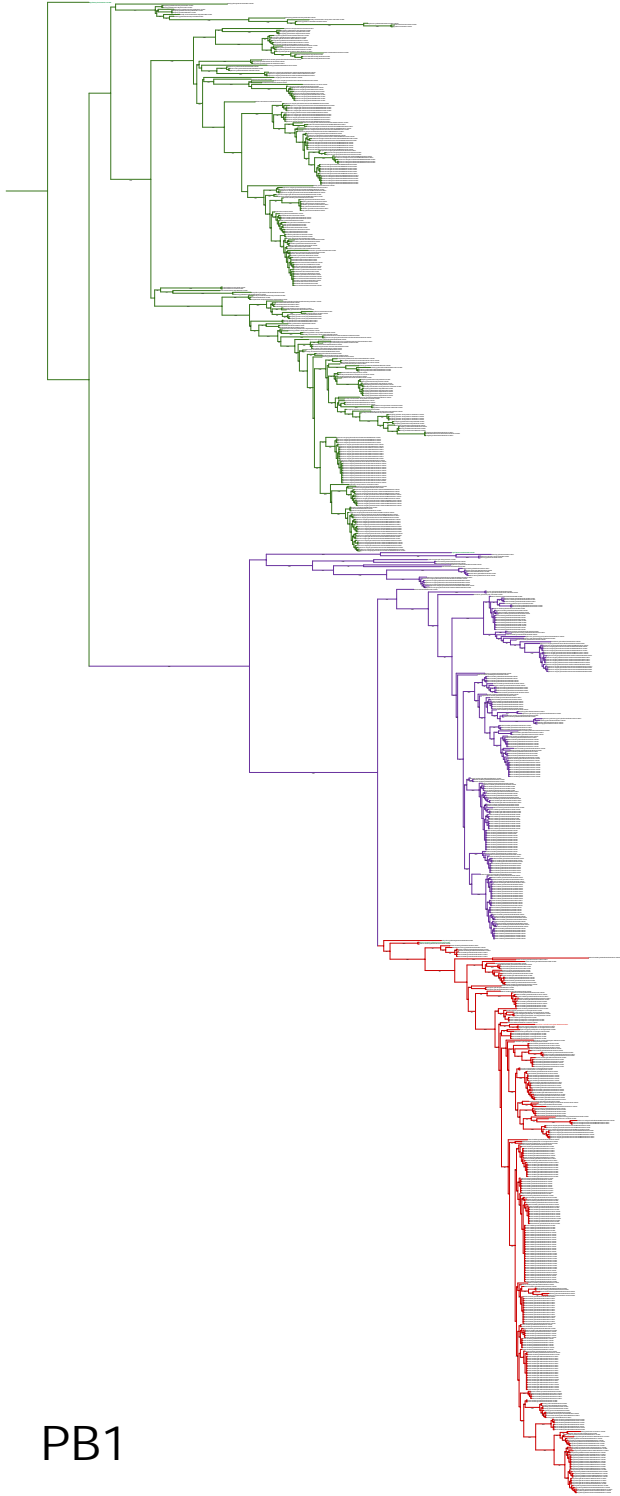

PB1

Supplement: Supplementary file 1 [file viruses-16-00329-s001.zip › Supplementary Figure S5.pdf]

Tree scale: 0.1

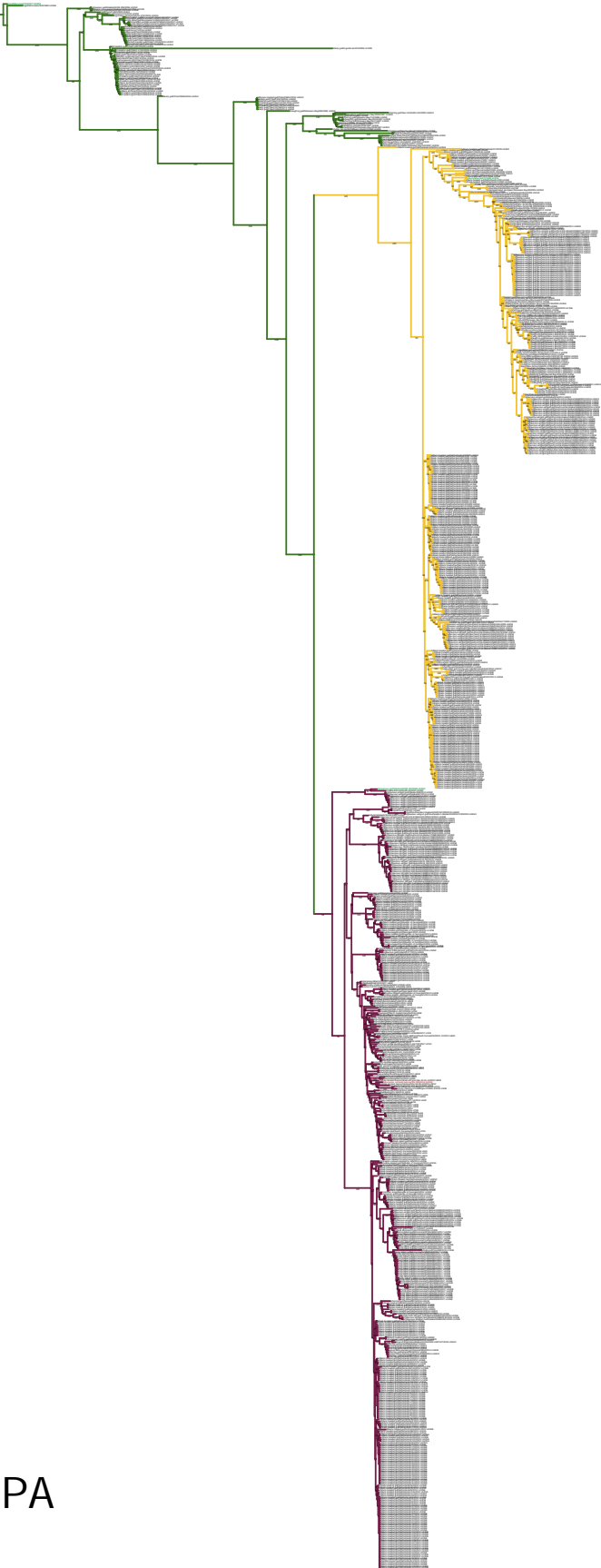

PA

Supplement: Supplementary file 1 [file viruses-16-00329-s001.zip › Supplementary Figure S6.pdf]

Tree scale: 0.1

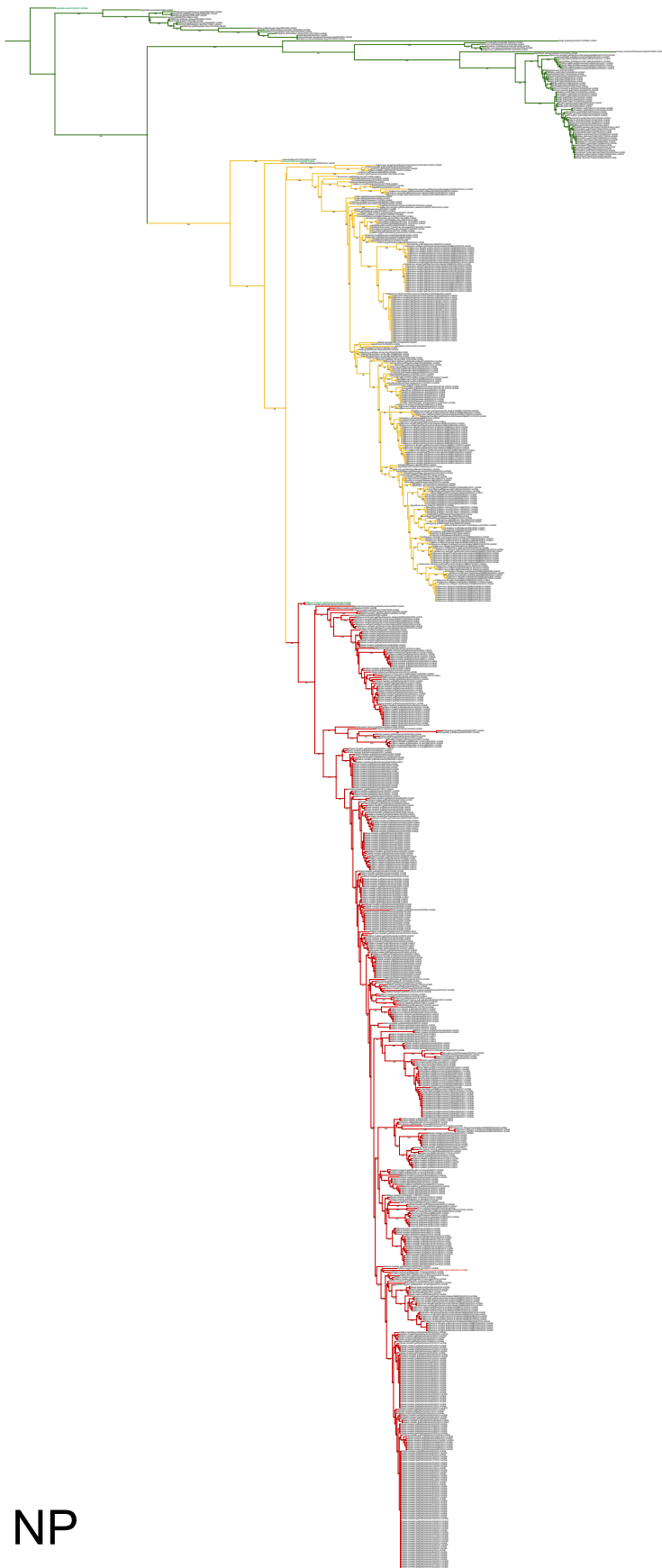

NP

Supplement: Supplementary file 1 [file viruses-16-00329-s001.zip › Supplementary Figure S7.pdf]

Tree scale: 0.1

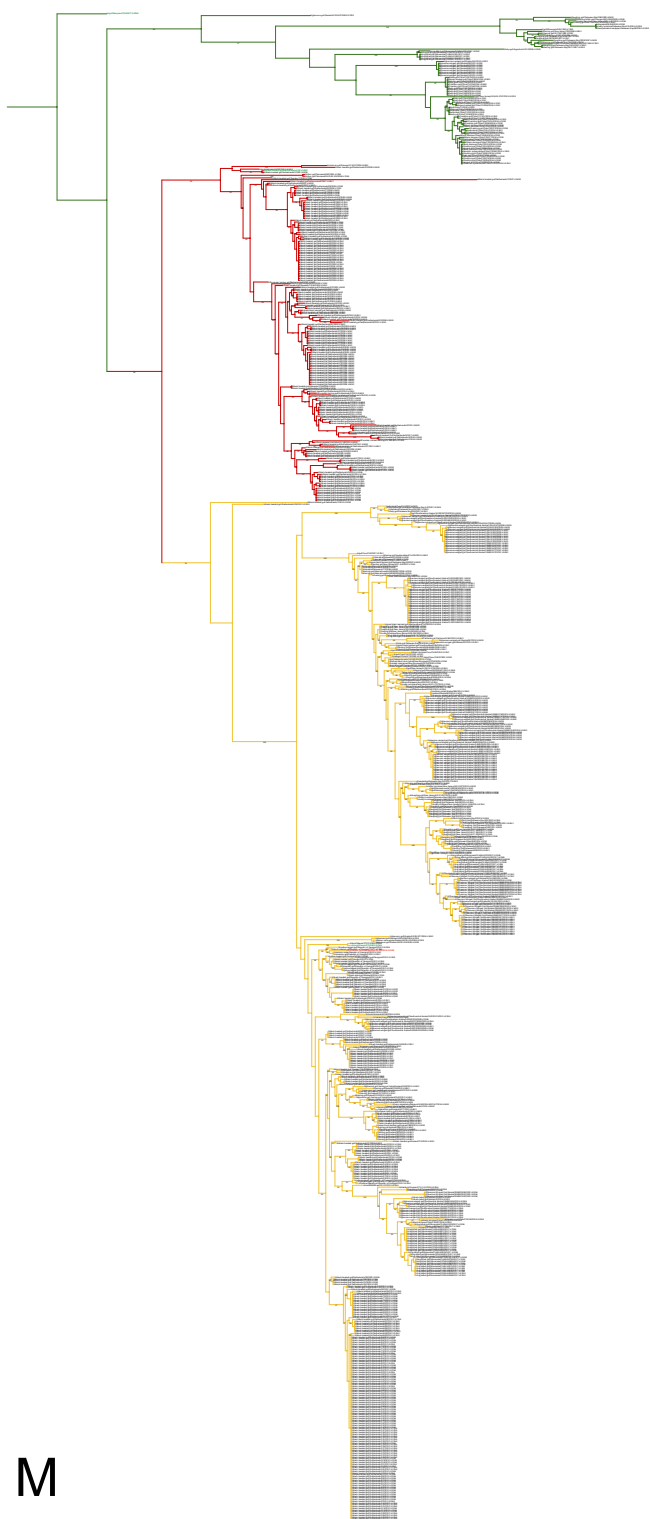

M

Supplement: Supplementary file 1 [file viruses-16-00329-s001.zip › Supplementary Figure S8.pdf]

Tree scale: 0.1

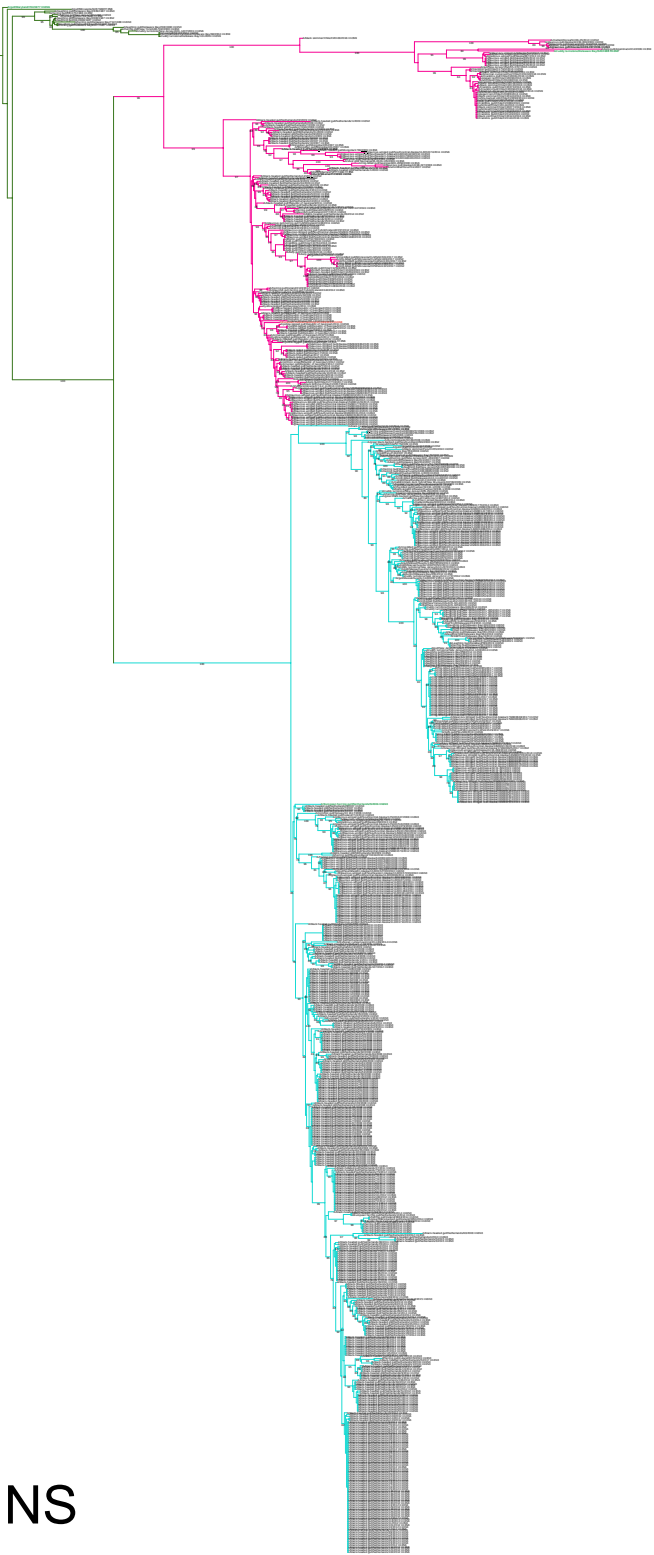

NS

Supplement: Supplementary file 1 [file viruses-16-00329-s001.zip › Supplementary Figure S9.pdf]
